# To the Editors of the Medical and Physical Journal

**Published:** 1801-12

**Authors:** John Phythian

**Affiliations:** Winchcombe, Gloucestershire


### *To the Editors of the Medical and Phyfical Journal.*

Gentlemen,

As the following fact tends to corroborate a pofition advanced by Dr. Jenner, namely, that the Variolous as well as Vaccine virus, after the puftules are in a declining ftate often parts with its fpecific properties, yet contains the power of exciting difeafe; I truft you will give it a place in your valuable publication.

In April laft, I inoculated three children of Mr. News, of Alftone in this neighbourhood, with Vaccine matter, which did not communicate the difeafe; and as they were expofed to the infection of the Small-pox, Mr. News wifhed to have them inoculated with that matter. At that period the only matter I could procure, was from the extremities of a child who had paffed the crifis of the difeafe two days, which I inferted into the arms of the three children. The two elder did not receive the infection, but the arm of the youngeft, a girl fifteen months old, inflamed and fuppurated. On one fide of the inoculated part two fmall puftules appeared fpontaneoufly, and the *child was indisposed*, fo that the parents and friends concluded fhe would be fafe from the difeafe in future. When the incifion firft inflamed, its appearance was very proper; but after the fuppurative ftage took place, I very much fufpected it was not characteriftic of the true difeafe, and accordingly inoculated this child and the other with Variolous matter. My fufpicion was verified, for the difeafe was communicated to this child as well as the other two, and they all paffed through its ftages with perfect fafety.

With refpect to another obfervation of Dr. Jenner's, that the Variolous is capable of bringing latent difeafes into action; I have long been fo fully perfuaded that fcrophulous affections have fucceeded the Small-pox, that I have invariably made this circumftance an argument in favor of the Cow-pox. Nor have I, fo far as my experience goes, ever feen maladies of any kind excited by the Cow-pox.

I am, &c.

JOHN PHYTHIAN.

*Winchcombe, Gloucestershire,*

*November* 15, 1801.

*Account*

